# Career self-efficacy as a mediator between career-specific parental behaviors and school career support on career doubt

**DOI:** 10.1186/s40359-024-01536-9

**Published:** 2024-01-19

**Authors:** Francis Cheung

**Affiliations:** Department of Psychology, Lingnan University, Hong Kong SAR, China

**Keywords:** Career doubt, Career self-efficacy, Career-specific parental support, School career support

## Abstract

This study evaluates whether career-specific parental behaviors and school career supports predict career doubt via the mediation of career self-efficacy. 227 participants aged between 18 and 25 completed an online questionnaire. Structural equation model (SEM) analysis showed that school career support was significantly related to career doubt via the mediation of career self-efficacy. However, the hypothesized mediation of career self-efficacy between career-specific parental behaviors and career doubt was not supported. Limitation and implications are discussed.

Emerging adulthood is a critical time for exploring life directions and forming a coherent sense of identity [1, 2]. The number of possibilities available for emerging adults triggers a sense of apprehension and skepticism about one’s identity and future [3]. This may lead to self-doubt and disrupt their ability to navigate the developmental tasks associated with this time of life. Emerging adults explore several key areas during this life stage, including work, romantic relations, and their worldviews [1]. One key challenge for these individuals is to resolve the confusion that originated from selecting their career path.

Making career decisions is difficult for emerging adults because it has a strong impact on their identity and well-being [4, 5]. Compared with children, emerging adults are more likely to describe their career choices as a dynamic interplay of their developmental stages and prevailing environmental circumstances [6, 7]. Bounded by limited career knowledge and/or actual work experience, emerging adults often encounter hesitation and worry, which may lead to self-doubt when choosing their future career. Career doubt is characterized by uneasiness and worry about one’s current career choice and the sense that others share the same feelings and ideas [8]. As a part of vocational identity, career doubt is common for emerging adults who are in the process of working towards career commitment. Career doubt could have a strong implication on one’s career decisions and behaviors, and research has been conducted to empirically test the association between career doubt and academic and career outcomes [10, 11]. Although past studies have examined developmental stages and personality as antecedents [11], studies that examine other career-specific antecedents that predict career doubt are limited.

Parents and schools represent two important agencies that contribute to individual career development [12, 13]. Drawing from the social cognitive career theory [14], this study aims to evaluate the association between career-specific parental behaviors and school career support (i.e., teacher and school support) in relation to career self-efficacy, which refers to an individual’s belief that he or she has the ability to complete the tasks related to making his or her career decision [15], and career doubt among emerging adults.

## Career doubt

Career doubt is “doubt, uneasiness, and worry about one’s current career choice and a sense that others share the same feelings and ideas” [8]. It is conceptualized as a consequence of the process of working toward a career commitment. Individuals who are doubtful about their career are less likely to take career action, such as career exploration and job search [10]. Although career doubt has clear implications for career behaviors, there is a paucity of studies that investigate factors that predict career doubt.

The present study adopts the SCCT to explore whether career-specific parental behaviors and school career support are associated with career doubt. SCCT is grounded in Bandura’s social-cognitive theory, it describes the formation of vocational interest, career choice, academic performance, and career performance. Based on the model, personal inputs (e.g., predisposition, ethnicity) and contextual factors (e.g., parental & school factors) affect an individual’s learning experience; this experience predicts one’s self-efficacy and eventually shapes his or her academic/career expectations. The model emphasizes the cognitive-person variable (e.g., career self-efficacy) and how it interacts with personal or contextual factors to shape the course of career development. Interestingly, when examining the role of contextual factors on career outcomes, prior studies primarily examine either the parental input [16] or school input [17, 18] on individuals’ career outcomes. However, there is a lack of studies that compare the relative importance of these two important contextual variables on career outcomes (i.e., career self-efficacy or career doubt). To address this research gap, this study explores the role of career-specific parental behaviors and school support predicts and their relationship with career self-efficacy and career doubt.

## Parental support and career self-efficacy

Parental influence is a powerful contextual determinant that affects career decisions and choices [19, 20]. To evaluate parental influence, past studies often used parent-child qualities, such as attachment, parenting styles, and family dysfunction [21, 22]. Career-specific parental behaviors provide an improved understanding of the underlying mechanism by which parents’ behaviors influence their children’s career decisions and development. In the present study, three career-specific parental behaviors are explored, namely parental support, parental influence, and lack of engagement.

Parental support refers to parents’ behaviors and assistance with their children’s career development. Similar to social support, parental support can be expressed in different ways. Some examples are parents letting their children make their own career choices while offering guidance, encouraging them to explore vocational interests and occupational options, and helping them reflect on their career choices. Parental support is a salient factor that contributes to their children’s career development. For example, parents’ vocational goals help their children reach their vocational goals through career modeling and career-related learning experiences [24]. The positive association between parental support and adolescent career development has been reported in different cultural contexts, such as Italy [20], the US [25], Korea [26], and China [27].

Career-specific parental behaviors are significantly related to their children’s career self-efficacy [28]. For example, parents could discuss career goals with their children to support their career choices and provide advice to them [29]. The evaluation of different options and parental guidance could help the younger adults make career choices and adjustments. Parental support could thus contribute to career self-efficacy by providing resources for navigating challenges as they explore career opportunities and helping children’s career exploration activities, or encouraging their children to pursue specific career goals [30]. These inputs will allow emerging adults to have higher confidence in understanding different career options and making decisions accordingly. Based on the above discussion, the present study hypothesizes that emerging adults who perceive higher career support from their parents will report higher career self-efficacy.

### Hypothesis 1a

Parental support is positively related to career self-efficacy.

## Parental interference and career self-efficacy

Parental interference is the perception of the pressure and control of parents over their children’s career decisions [23]. Shared understanding between parents and emerging adults’ career goals and aspirations is challenging because it depends heavily on mutual understanding of each other’s preferences and communication [16]. Thus, these parties often differ on the amount of influence they believe parents should have in making important career decisions [31, 32]. For example, with more actual work experience, parents often serve as important vocational advisors for their children. They may engage intentionally in various actions to facilitate their children’s career development [33] and to develop the kind of lives they would like their children to live [34]. Some parents may want to implement or enforce their ideas about their children’s occupations, regardless of their children’s wishes and decisions [33]. Thus, adolescents in these families are generally more passive in the process of career preparation.

Parental involvement is crucial to the development of adolescents and young adults’ career outcomes. However, in some cultures (e.g., collectivistic) where adolescents have to show respect and filial piety by listening to their parents, parental interference should be considered autonomy-thwarting and would be negatively related to adolescents’ career efficacy because adolescents would likely feel that they have to comply with the parents’ agenda [35]. This decision may not necessarily correspond to their own needs. Interestingly, a recent research study highlighted the importance of an appropriate degree of support and non-manipulative interference as important factors to determine whether parental support is effective in supporting their children’s career outcomes. In particular, when parents are “over-willing” to provide support and when their intentions are manipulative and not genuinely supportive of freedom of choice, such a high level of involvement could not help their children build career confidence; rather, it would lead to higher sense of confusion. Furthermore, those who perceive that their parents’ thinking has been pushed on them may experience unnecessary confusion and difficulty when making career choices, which may hinder their career development [36]. In fact, Fan et al. [37] reported that a higher level of family intrusiveness was a significant predictor of career decision-making difficulties among university students in Hong Kong and the US. Based on this idea, the following hypothesis is formulated:

### Hypothesis 1b

Parental inference is negatively related to career self-efficacy.

## Lack of parental engagement and career self-efficacy

Lack of parental engagement is the third career-specific parental behavioral dimension in predicting career self-efficacy. It refers to the perception that an individual’s career development process is being neglected by their parents. Parental engagement and encouragement are important because they have a direct impact on children’s learning experiences, self-efficacy, and outcome expectancy [38]. Parents also help their children shape their career values, interests, and skills for their future careers [39]. However, some parents do not actively help their children make career choices or support their career development [13]. According to Guan et al. [40], the lack of parental engagement poses a challenge for adolescents and young adults because they may not have sufficient resources to solve their problems in career exploration and decisions. A lack of parental engagement has an adverse impact on adolescents’ career development and future career trajectory [13]. Without the resources of their parents, emerging adults may not be able to reconcile the conflict when making career choices, which inevitably lowers their sense of career self-efficacy. Based on the above discussion, the following hypothesis is proposed:

### Hypothesis 1c

Perceived lack of engagement is negatively related to career self-efficacy.

## Teachers’ support and career self-efficacy

According to the SCCT, school, as a distal factor, has an important impact on the career development of adolescents. Two aspects of school-related support are considered in this study, including teacher support and school career training. The importance of teachers’ support for students’ career development has been discussed in various theories, including ecological system theory [41], career construction theory [42], and social cognitive career theory [14]. Supportive teachers provide students with information on the current job market and encourage them to engage in exploration [43]. These teachers also suggest where students can find more information about a particular occupation. Through career role models and skills development opportunities, teachers can enrich their students’ learning experiences, which would indirectly contribute to students’ sense of self-efficacy [44]. Similar to parents, supportive teachers could share their values and attitudes that may influence the career preferences and confidence of students. They could provide students with the necessary assistance for making career-related decisions and help them understand the different career options available. Therefore, emerging adults who perceive support from their teachers should feel confident in making career-related decisions and adjustments.

### Hypothesis 2a

Teachers’ support is positively related to career self-efficacy.

## School support and career self-efficacy

While teacher support for students’ career development may be relational (i.e., social or emotional), school career support is considered more instrumental [45, 46]. Schools implement different strategies and interventions to support students’ career development [47]. Based on the Dykeman et al., taxonomy, career development intervention programs can be categorized into four major clusters [48]. The first cluster is work-based career intervention, where students acquire career knowledge through actual participation. Some examples of work-based career development programs include internships and job shadowing. The second cluster is advising, where intervention is designed to provide students with direction and resolve their uncertainty via activities like career peer advising, tutoring, or career counseling. The third category is introductory taxonomy, with the aim of awakening students’ interest in and requirements for a particular career. Intervention programs under this category include career days, career fairs, and career field trips. Finally, schools can also opt for curriculum-based intervention to promote knowledge and skills that are essential to the world of work. Career and technical education courses, skill-based training, and career academy courses fall into this category.

Although school support can be expressed in idiosyncratic ways, these supports are important to aid adolescents’ career development [49]. For example, career information is crucial for students to make career choices [50]. Schools that provide career counseling or assessment help their students assess and evaluate their career interests and strengths. The provision of such career-related activities undoubtedly helps students understand the career options available and enhances their confidence to make relevant career choices. For example, in a sample of poor youth of color in the US, instrumental school support was found to be directly related to vocational expectations [50]. Thus, when schools provide a lot of practical career-related information, adolescents and young adults gain confidence in making their career decisions, given that more resources are available. Based on these findings, the following hypothesis is formulated:

### Hypothesis 2b

School support is positively related to career self-efficacy.

## Career self-efficacy and career doubt

The present study hypothesizes that individuals with high career self-efficacy should report low career doubt. In particular, individuals with high career self-efficacy have high levels of confidence to engage in tasks associated with making career decisions and committing to a career [14]. Individuals with high career self-efficacy also tend to report high career preparation [51] and exploration [52]. Given that individuals with high career self-efficacy have strong beliefs about making effective career decisions [53], they do not feel anxious, or doubtful about their career decisions. Moreover, given their perception that they can handle career decisions and related matters, they would not worry about how others perceive their career decisions. Therefore, career self-efficacy can be seen as a valuable personal resource during the uncertain time of a career search. It allows them to make career decisions and be confident about those decisions. Thus, the following hypothesis is formulated:

### Hypothesis 3

Career self-efficacy is negatively correlated with career doubt.

Summarizing the earlier sections, parental behaviors and school career support predict career self-efficacy, and the latter is hypothesized to be significantly related to career doubt. In other words, career self-efficacy is positioned as a mediator between distal factors (i.e., career-specific parental behaviors and school supports) and career doubt. Thus, the following hypothesis is formulated:

### Hypothesis 4

Career self-efficacy mediates the relationship between perceived career-specific parental behaviors and school career support for career doubt.

## Method

### Procedure and participants

Participants were recruited online through Mturk in April 2020. A total of 701 online samples were included. Screening for eligibility was done by MTurk. To be eligible for the study, participants had to be 18 years of age or older, working full-time, and residing in the U.S. They had to have previously completed at least 1000 tasks with a 99% approval rating or above. To ensure the quality of feedback, two attention checks were inserted. 19 participants either failed to respond to the attention checks correctly or did not complete the entire questionnaire. Consequently, the total valid sample was 682. Among them, 41 (6%) of participants aged between 18 and 21, 189 (27.7%) aged between 22 and 25, and 452 (66.3%) aged between 26 and 30. Since the developmental tasks for adolescents and young adults may be different, and the effect on parents and schools should be more relevant to a younger population, the analysis thus included only emerging adults (aged between 18 and 25). As a result, only 229 participants were included in the study. Among them, 97 (42.4%) were male and 128 (55.9%) were female. 72.2% had a full-time job or were working a part-time job. Ethical approval was obtained from the university ethical review committee, which the PI is affiliated with. Participants were asked to complete an informed consent form before they began to complete the online survey. They were given a unique completion ID number for participation fee claims. Participants were given USD 1.75 after successfully completing the survey.

### Measures

#### Career-specific parental behaviors

Parental behaviors were measured by the career-specific parental behavior scale [23]. This scale consists of three subscales, namely parental support (5 items), parental interference (5 items), and lack of engagement (5 items). Sample items include “my parents talk to me about my vocational interests and abilities” (parental support), “my parents have their own ideas about my future vocation and try to influence me accordingly” (parental interference), and “my parents are not really interested in my future vocation” (lack of engagement). These scales were significantly correlated with adolescents’ career exploration [23]. Participants were asked to respond on a Likert scale, ranging from 1 (“strongly disagree) to 5 (“strongly agree). The alpha coefficients were.90,.90, and.82 for parental support, parental interference, and lack of engagement, respectively.

#### School support

The school support scale was measured by the subscale developed by Diemer [50]. The scale consists of four items; a sample item includes “my school offers interest inventories”. This scale was found to be significantly correlated with vocational expectations (Diemer, 2007). Participants were asked to respond on a Likert scale, ranging from 1 (“strongly disagree) to 5 (“strongly agree). The alpha coefficient of the scale was.77.

#### Teacher support

Teachers’ support was measured by the teacher support scale proposed by Farmer et al. [54]. The scale consists of six items; a sample item includes “teachers in my school don’t care about my future career plans” (reversed score). This scale was significantly related to educational plans and career expectations in an earlier study. Participants were asked to respond on a Likert scale, ranging from 1 (“strongly disagree) to 5 (“strongly agree). The alpha coefficient of the scale was.74.

#### Career self-efficacy

Career self-efficacy was measured by the career self-efficacy scale proposed by Taylor and Betz [15]. The scale consists of 25 items and captures five dimensions, including self-appraisal (e.g., accurately assess your abilities), occupational information (e.g., use the internet to find information about occupations that interest you), goal selection (e.g., select one occupation from a list of potential occupations that you are considering), making plans for the future (e.g., identify employers, firms, and institutions relevant to your career possibilities), and problem-solving (e.g., change occupations if you are not satisfied with the one you enter). The alpha coefficients were 0.85, 0.77, 0.86, 0.84, and 0.82 for self-appraisal, occupational information, goal selection, making plans for the future, and problem-solving, respectively.

#### Career doubt

Career doubt was measured by the five-item self-doubt sub-scale of vocational identity status assessments [8]. A sample item included “I may not be able to get the job I really want”. The scale was significantly related to academic and career coping variables [9]. Participants were asked to respond on a Likert scale, ranging from 1 (“strongly disagree”) to 5 (“strongly agree”). The alpha coefficient of the scale was 0.85.

#### Demographics

Participants were also asked to indicate their gender, age, and annual income.

### Analysis

Descriptive statistics, internal consistency of measurements, missing data analysis (Little’s missing completely at random, MCAR test) and inter-correlations were first conducted with SPSS. Pairwise deletion was adopted to handle missing cases. Structural equation modeling (SEM) using the JASP computerized program was performed to test the hypothesized model. In particular, lack of engagement, parental interference, and parental support were used as the observed indicators of the latent factor of “career-specific parental behaviors”. School support and teacher support were indicators of the latent factor of “School career support”. Finally, planning, problem solving, occupational information, goal setting, and self-appraisals were used as the observed indicators of the latent factor of “career self-efficacy”. Chi-square analysis, Root Mean Square Error of Approximation (RMSEA), Normed Fit Index (NFI), and Comparative Fit Index (CFI) were used to evaluate the model fit. The maximum likelihood solution was adopted as the estimation procedure, and raw scores were used as the data input.

In the hypothesized framework, a full mediation model is proposed as the effects of career-specific parental behaviors and school factors will first predict career self-efficacy and subsequently, to career doubt. However, both career-specific parental behaviors and school-related factors [38, 40] were found to be directly related to career outcomes of adolescents and young adults. Considering the potential direct effect of career-specific parental behaviors and school career support on career doubt, direct paths from these latent factors to career doubts were added. In other words, an alternative model (i.e., partial mediation effect model) will be introduced and chi-square difference test will be used to compare the overall model fit of the original and alternative models.

## Results

### Descriptive statistics and correlation

The alpha coefficients, means, standard deviations, and inter-correlations among the study variables are presented in Table 1.

**Table 1:** Descriptive statistics and correlation

	1	2	3	4	5	6	7	8	9	10	11
1. Parental support	(0.90)										
2. Parental interference	0.14*	(0.90)									
3. Lack of engagement	− 0.69**	− 0.01	(0.82)								
4. Teacher support	0.35**	− 0.16*	− 0.37**	(0.74)							
5. School support	0.30**	− 0.02	− 0.24**	0.41**	(0.77)						
6. CSE-self appraisal	0.21**	− 0.15*	− 0.22**	0.45**	0.35**	(0.85)					
7. CSE-occupational information	0.26**	-0.10	− 0.21**	0.49**	0.44**	0.76**	(0.77)				
8. CSE-goal selection	0.24**	− 0.16*	− 0.20**	0.50**	0.38**	0.85**	0.76**	(0.86)			
9. CSE-making plans for the future	0.25**	-0.12	− 0.19**	0.47**	0.44**	0.80**	0.77**	0.83**	(0.84)		
10. CSE-problem solving	0.22**	− 0.08	− 0.18**	0.46**	0.37**	0.79**	0.74**	0.79**	0.84**	(0.82)	
11. Career doubt	− 0.15*	0.34**	0.22**	− 0.50**	− 0.16*	− 0.53**	− 0.45**	− 0.59**	− 0.55**	− 0.48**	(0.85)
Mean	3.07	2.46	2.34	3.66	3.78	3.47	3.59	3.34	3.36	3.25	2.82
Standard deviation	1.11	1.11	0.94	0.67	0.89	0.77	0.75	0.84	0.86	0.79	1.01
Range	1–5	1–5	1–5	1–5	1–5	1–5	1–5	1–5	1–5	1–5	1–5

*Notes*: *N* = 227. Reliabilities on the diagonal with parentheses. *p <.05; **p <.01

### Structural equation model analysis

The chi-square value of the proposed model was 126.82 (*df =* 41, *p <*.01), and the RMSEA was.10 (90% CI: 0.08 and 0.12). Other fit indices were above the.90 criterion (NFI = 0.92; CFI = 0.95). Taken together, these model fit indices suggested that there was a reasonably good fit between the data and the proposed model. In terms of path estimates, school career support was significantly related to career self-efficacy (coefficient = 1.47, *z* = 3.60, *p* <.01), however, career-specific parental behavior was not related to career self-efficacy. Career self-efficacy was negatively related to career doubt (coefficient = − 0.37, *z* = -5.79, *p* <.01). The examination of indirect effect showed that career-self efficacy mediated the relationship between school career support and career doubt (coefficient = − 0.54, *z* = -5.61, *p* <.01). However, the mediation of career self-efficacy between career-specific parental behavior and career doubt was not significant (coefficient = 0.15, *z* = 1.85, *p* =.06).

### Testing a partial mediation model

**Fig. 1:**
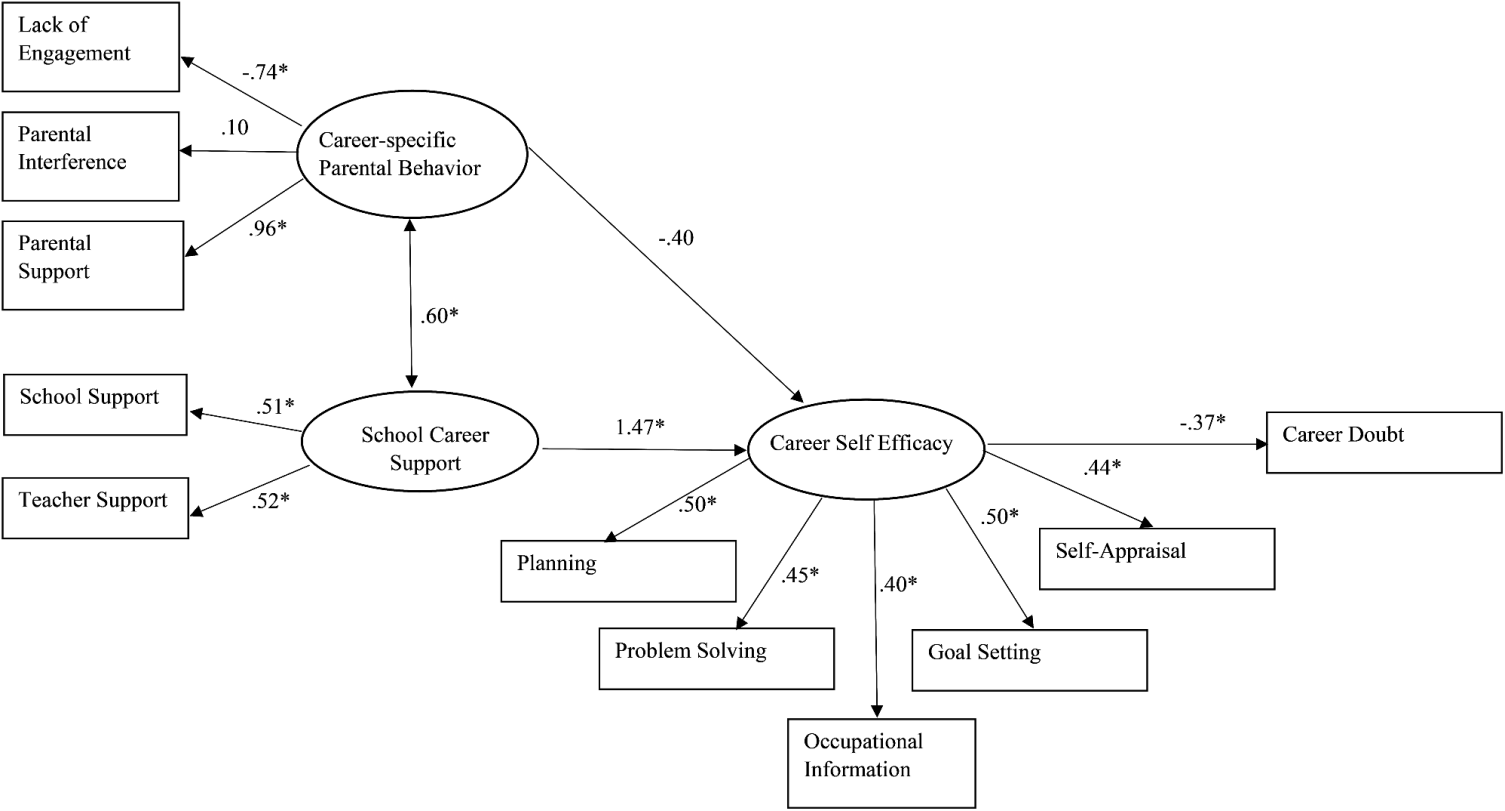
Parental and school career support on career doubt by social cognitive career theory finalized model with standardized parameters

Chi-square difference test was performed to evaluate whether the alternative model provides better fit to the data. In particular, two additional paths (Career-specific parental behaviors and School career supports to career doubt) were added. Results showed that the chi-square was not statistically different from the original full mediation model (∆χ^2^ = 1.25, *p* >.05). In other words, the inclusion of the direction paths from career-specific parental behaviors and school career support to career doubt did not significantly improve the overall model fit. Therefore, the parsimonious full mediation model was adopted. Details of model comparisons and the path estimates of final model are presented in Table 2 and Fig. 1, respectively.

**Table 2:** Structural equation model comparison

Model	χ^2^	Degree of freedom	NFI	CFI	RMSEA (90% CI)
Model 1. Full mediation model	126.82	40	0.92	0.95	0.10 (0.08–0.12)
Model 2. Partial mediation model	125.57	41	0.92	0.95	0.10 (0.08–0.12)

*Note*: NFI = Bentler-Bonett Normed Fit Index; CFI = Comparative Fit Index; RMSEA = Root Mean Square Error of Approximation

## Discussion

Based on the SCCT, this study aims to examine whether career-specific parental behaviors and school career support predict career self-efficacy and career doubt among emerging adults. SEM results suggested that school career support was significantly related to career doubt via the mediation of career self-efficacy. However, the hypothesized mediation effect of career self-efficacy between career-specific parental behaviors and career doubt was not supported.

### Career-specific parental behaviors, career self-efficacy, and career doubt

The results of this study provide a mixed picture of the relationship between career-specific parental behaviors and career outcomes. In the correlation analysis when bivariate relationships were tested, individual career-specific parental behavior dimensions, including parental support and lack of engagement, were significantly related to career self-efficacy facets. These findings are in line with existing studies [30] that showed parents play an important role in their children’s career development and confidence in making career choices. However, the SEM model shows that career-specific parental behaviors as a latent factor was not significant in predicting career self-efficacy, especially when school career supports were considered simultaneously. From a developmental perspective, the importance of parents influencing their children drops [55, 56], especially when many of the emerging adults choose to move away from their parents and live independently. The importance of parental role in their children’s career outcomes might thus decline. Besides, emerging adults may have more exposure to the actual job market through moving between different part-time or full-time jobs [1, 2] or they may learn from their peers who have other work exposure. These work experiences may provide more relevant input to establish their career self-efficacy and confidence [57] over the persuasion from their parents. The seemingly incoherent findings of career-specific parental behaviors on career outcomes could be explained by a recent study [36]: instead of adopting the variable-center approach (e.g. correlation/regression analysis), the researchers team examined how different combinations of these parental behaviors relate to career outcomes. In particular, based on cluster analysis, they reported that adolescents with parents who showed moderate levels of support and low levels of interference (i.e., willing parents) indicated a lower level of identity diffusion, made fewer help requests, and had lower choice conflicts. However, when parents are overly supportive and frequently intervene in their children’s choices (i.e., involved parents), adolescents would usually report a higher sense of identity diffusion and have higher choice conflict. Thus, when considering the impact of career-specific parental behaviors on adolescents’ career outcomes, it may be more fruitful to think in terms of how different combinations of career-specific parental behaviors shape the adolescents’ career behaviors. For example, when parents provide a high level of support to their children but at the same time they intervene too much, the positive effect of parental support may be weakened since their children may find the support unnecessary. However, when parents provide an appropriate level of support without giving their children too much intervention, it should be optimal to support the adolescents in making their own career choices, reducing their career doubt, and helping them develop career commitment in the long run.

### School supports, career self-efficacy, and career doubt

Compared to career-specific parental behaviors, school career support is considerably more consistent in relating to emerging adults’ career self-efficacy. As discussed earlier, teachers and schools provide different types of assistance to students. Teachers as role models could readily provide resources to support their students’ career self-efficacy and reduce their career doubt. Schools could support the emerging adults’ career outcomes via curriculum design and structured career support (e.g. career interest assessment and counseling, job exhibition). With both the individualized teacher support and the systematic school programs, these measures would undoubtedly strengthen individuals’ sense of control and reduce their doubts in formulating their career plans.

### Theoretical contributions

This study makes several contributions to career literature. First, it advances the understanding of the antecedents of career doubt among emerging adults. Despite the seemingly logical association between career doubt and career self-efficacy, no study has empirically investigated the association between the two. This is the first study to adopt the SCCT to critically evaluate this association. Moreover, since the majority of the studies examined the importance of either parental behaviors [16] or school factors [17, 18] to support career outcomes, studies that examine and compare the relative importance of parental and school support in relation to career doubt are scarce. This study evaluates the relative importance of contextual and individual factors and thus fills a research void on career doubt. In sum, this study helps delineate the role of parents and school on career self-efficacy and career doubt. These findings can further enrich the existing literature on how contextual factors relate to career outcomes among emerging adults.

### Practical implications

Career doubt hinders individuals’ career exploration and development. Therefore, locating ways to mitigate such career uneasiness and doubt is vital. This study provides insights on how to achieve this goal by strengthening career self-efficacy. This study reaffirms the importance of school career support for emerging adults’ career self-efficacy. Having strong career self-efficacy could encourage them to explore different career options and be intrinsically motivated. To strengthen students’ sense of career self-efficacy, teachers may use their experience or invite alumni or peers to share their successful career search experiences with students. Such sharing could strengthen students’ belief in their abilities. They could also provide students with feedback throughout their career exploration or motivate them through verbal persuasion [58]. Schools can support career development via curriculum design and the provision of career-related support, such as career assessment and counseling. The overall goal is to provide reflection and insights for emerging adults to better understand their strengths, weaknesses, and career interests. Schools could also organize placement or job talks to keep them abreast of the latest human resource market developments. With information on oneself and the general labor market, students would feel more confident in making career choices, which would eventually lower their career doubt. The engagement of placement will provide unique learning experiences and social environments for the adolescents, and such stimulation will foster career identity change, which consequently lead to higher career resources (e.g., career self-efficacy, job knowledge) and actual career outcomes (e.g., finding employment more quickly). To achieve this goal, schools and universities should actively locate organizational partners that can provide high-quality placement opportunities that emphasize autonomy and task identity for students to participate in.

Finally, parents should find a good balance between support and intervention. Although parents are eager to provide support to their offspring, actively pushing their ideas and values on their children may not be a good idea, even though such action is out of concern. Parents who are overly involved tend to result in higher confusion; this finding is partly due to the fact that parents might have suggested an excess of alternatives [36]. Besides, in order to enhance parental input and strengthen their adolescents’ career efficacy, parents should acquire more career-related information and skills that could facilitate their discussion with their adolescent. For example, by working with professional career counselors, parents could learn career-related communication skills in order to provide verbal feedback about their adolescents’ career choices and work-related skills [25, 59].

### Limitations and future study

This study has several limitations, and its results must be interpreted with caution. First, this study adopts a cross-sectional, self-administered online survey for data collection. This approach does not allow for the delineation of cause and effect among distal factors (i.e., career-specific parental behaviors and school support), career self-efficacy, and career doubt. Moreover, common method variance may affect observed associations [60]. Future studies should obtain data from other sources (e.g., parents and school’s data) for external validation.

Second, school support can be categorized into four major categories: work-based, advising, introductory, and curriculum-based intervention. In this study, the assessment of school support was primarily focused on advising and introductory support; work-based and curriculum-based support were not included. In order to obtain a more holistic picture of how school career support relates to emerging adults’ career development, future studies should consider the inclusion of other intervention strategies and test their relation to career development.

Third, this study only limits its focus to whether family and school factors could influence career efficacy and doubt among American adolescents and young adults. Parents and family are important agents of socialization that shape adolescents’ sense of career goals and efficacy through role modeling and the sharing of parental beliefs and expectations [61]. However, the expression of parental behaviors and parent-child interactions is different across different cultural contexts (Eastern and Western). In Chinese culture, family is the primary social unit where adolescents and young adults are expected to listen to and follow parental advice in order to show “filial piety”. Against this cultural backdrop, parental behaviors might have a stronger influence on their children’s career development [61, 62]. In a cross-cultural study which compared how parents, schools, and peers contribute to the sense of career self-efficacy among adolescents in Hong Kong, Shanghai, and the US, results showed that adolescents in Hong Kong indicated lower support from parents and peers in contributing to career efficacy when compared to their Shanghainese and American counterparts [62]. Thus, before drawing a conclusion on the role of parental behaviors on career self-efficacy and career doubt, future studies may adopt a cross-cultural design to evaluate whether the career-specific parental behaviors are effective or ineffective in predicting career self-efficacy and career doubt in different cultures.

Finally, the focus of the paper has been limited to the contextual factors in shaping career self-efficacy and career doubt. Based on the SCCT, individual factors, such as predispositions, gender, health status, and race or ethnic identity, also play an important role in one’s career development. As discussed earlier, identity has been closely related to the development of the career identity of an individual. In a study conducted with South-East Asian adolescents in Hong Kong, adolescents who have a clearer sense of identity, such as higher affirmation and belonging to their ethnic group, identity achievement, and ethnic behaviors, tend to also have higher career expectations of themselves [63]. Similarly, the sense of ethnic identity was positively related to the sense of self-efficacy, which in turn contributed to the outcome expectations for career choices [64]. Thus, future studies should expand the scope of the present study to examine the interplay of both personal and contextual factors in relation to the career development of emerging adults.

## Conclusion

This study examined two salient contextual factors, namely career-specific parental behaviors and school career supports, in relation to emerging adults’ career self-efficacy and career doubt. In sum, school career support is a more salient factor in predicting career self-efficacy, and the latter is a significant mediator between school career support and career doubt.

## Data Availability

The datasets generated during and or analyzed during the current study are available from the corresponding author on reasonable request.
